# A Case Report of Dacrystic Seizures in the Psychiatric Emergency Services Department

**DOI:** 10.7759/cureus.23632

**Published:** 2022-03-29

**Authors:** José C Medina, Roxana Galván, César Y Garfias, Diana J Arteaga

**Affiliations:** 1 Teaching Department, National Institute of Psychiatry, Mexico City, MEX; 2 Psychiatric Emergencies Department, National Institute of Psychiatry, Mexico City, MEX

**Keywords:** focal motor seizure, neuropsychiatry symptoms, emergency service, crying, seizure classification

## Abstract

Epileptiform syndromes are represented by a variety of clinical scenarios. In this context, dacrystic seizures (DS) are characterized by paroxysmal episodes of stereotyped crying and are considered a rare ictal phenomenon. Their neuroanatomical and pathophysiological findings tend to be nonspecific, and to date, there is no consensus on their treatment. Additionally, most of the existing case reports describe that the patients who suffer from them are usually refractory to conventional care. Also, most of the existing literature is approached from a neurological practice perspective; however, there's evidence of patients with these paroxysms that occasionally end up in the hands of psychiatric services due to the uniqueness of their symptoms. However, there is very little information about this phenomenon due to its rarity. For this reason, this manuscript presents the case of a middle-aged man with these seizures who initially attended a psychiatric emergency service and subsequently received neuropsychiatric management and follow-up.

## Introduction

Dacrystic seizures (DS) are a type of epileptiform phenomenon characterized by paroxysmal crying [1]. Their characteristics include stereotyped weeping, frowning, and a subjective feeling of distress in the absence of a depressed mood [2]. The paroxysms are brief, lasting up to 10 to 20 seconds long, may occur several times a day [3]. These are thought to be a rare type of seizure, literature about them is scarce, and their prevalence has not been accurately measured [4]. Up to this point, most cases that have been reported are isolated incidents or small case series [5]. Attempts to correlate the clinical presentation of DS with a specific etiology have been previously made [6]. For instance, the majority of reported paroxysms seem to begin with focal seizures that are then localized to the frontotemporal regions and subsequently lateralized to the nondominant hemisphere [7]. The nature of the lesions that cause them remains unclear given the fact that a minor proportion of patients will lack anatomical abnormalities, but the majority of the reported seizures appear to be triggered in the hypothalamus and seem to be frequent in individuals with hypothalamic hamartomas [8]. Blumberg et al. also agreed that the underlying neuropathology may be located in the temporal cortex [9]. Furthermore, DS seem to evolve as the person gets older and may spread and affect either side of the brain, leading to absence, atonic, tonic, and tonic-clonic seizures [9,10]. Diagnosis of these might be a difficult endeavor since symptoms are typically missed or are not initially regarded as seizures [11]. Additionally, electroencephalographic tests often give normal findings, but some may display readings that range from subtle alterations of the basal brain rhythm to frank abnormal results [9]. Such changes are not easily identified, and these might require periodic testing to detect the seizures [1]. Regarding its treatment, patients are often described as refractory to usual epileptic management once diagnosed with DS, and the overall reported prognosis tends to be poor [9]. Notwithstanding the direct and obvious relationship of these type of seizures in conjunction with neurological medical practice, there are reports of patients with this condition who initially attend to psychiatric services due to the specific presentation of their symptoms [12]. Herein, we report an instance of a 60-year-old male who presented with DS in the setting of a psychiatric emergency services department while complying with the CARE Case Reports Guideline [13].

## Case presentation

Patient information

The present case deals with a 60-year-old male who went accompanied by his son to the psychiatric emergency service of a public institution with the referred reason for consultation of “uncontrollable crying.” Regarding his background, he mentioned having finished his basic education, being a masseur by trade, being single and catholic. Regarding his family history, he mentioned having a genetic load for type 2 diabetes and systemic arterial hypertension in his maternal line, and he denied any type of hereditary psychopathological family history in any of his paternal or maternal branches. Regarding his personal history, he reported having a diagnosis of systemic arterial hypertension of approximately one year of evolution, treating it with Enalapril 10/day, and denied any other history of allergies, surgeries, trauma, or transfusions. Concerning his mental health history, he described that about a month ago he had begun psychiatric care in a non-governmental institution, mentioning that he was told that to date he had a diagnosis of “depression and anxiety under study” and that he was started on pharmacological treatment with Citalopram 20mg/day, Quetiapine 25mg/night and Clonazepam 0.5mg/night, and denied having presented any type of adverse effect associated while taking them. When questioned about the use of these medications according to what was prescribed by his psychiatrist, he admitted to using up to 2mg/night of Clonazepam on occasions, justifying the decision as he remarked that his symptoms appeared to decrease in frequency with this dose. However, the patient later clarified that he had not identified considerable relief from them in a month-long treatment with these drugs. Additionally, he denied having done any type of non-suicidal self-injurious behavior, having made suicide attempts and categorically rejected having experienced situations of violence of any kind or psychological trauma. Regarding the use of psychoactive substances, he also denied the use of these and only reported being a consumer of alcohol in social circumstances, approximately twice a year, and in unspecified amounts.

Clinical findings

In terms of his illness, he describes that it began approximately five months prior to attending this service, and without an apparent stressor. He mentioned that, unexpectedly, he began to wake up earlier than usual while identifying idiopathic abdominal paresthesias that last for several seconds and then subside. He mentioned that this sensation is uncomfortable enough to wake him up and that after their disappearance, he presents idiosyncratic urgency and the need to cry, later resulting in paroxysmal episodes of intense crying, accompanied by lacrimation, stereotyped panting, and a subjective feeling of discomfort. The patient and his son reported that these episodes persist for a short time, not exceeding one minute in most cases, but that they progressively have been occurring more than 50 times per day. Additionally, they denied that these paroxysmal episodes were accompanied by changes in alertness, muscle tone, strength, or any other symptom as a whole, but they stated that the paroxysms had already begun to affect his ability to perform his job and other daily activities due to their increasing frequency. The patient referred feeling “perplexed” regarding the appearance of these symptoms, arguing that initially he suspected that they were due to “accumulated stress,” but that he gradually began to doubt if “he had a psychiatric problem” due to the persistent crying, which is why he said he decided to initiate care in a psychiatric service. Also, the patient and his relative confirmed that he had not presented depressive, manic, psychotic, or obsessive symptoms. Additionally, he denied presenting suicidal ideation, self-harm, or any other risk behavior. He only reported disproportionate concern about his current state of health since the onset of the symptoms already described, and that this was accompanied with localized muscle tension in his neck and shoulders, symptoms that the patient associated with little restful sleep. In addition, no history of other ictal symptoms or paroxysms of different characteristics were identified.

Timeline, diagnostic assessment, and clinical outcomes

**Figure 1:**
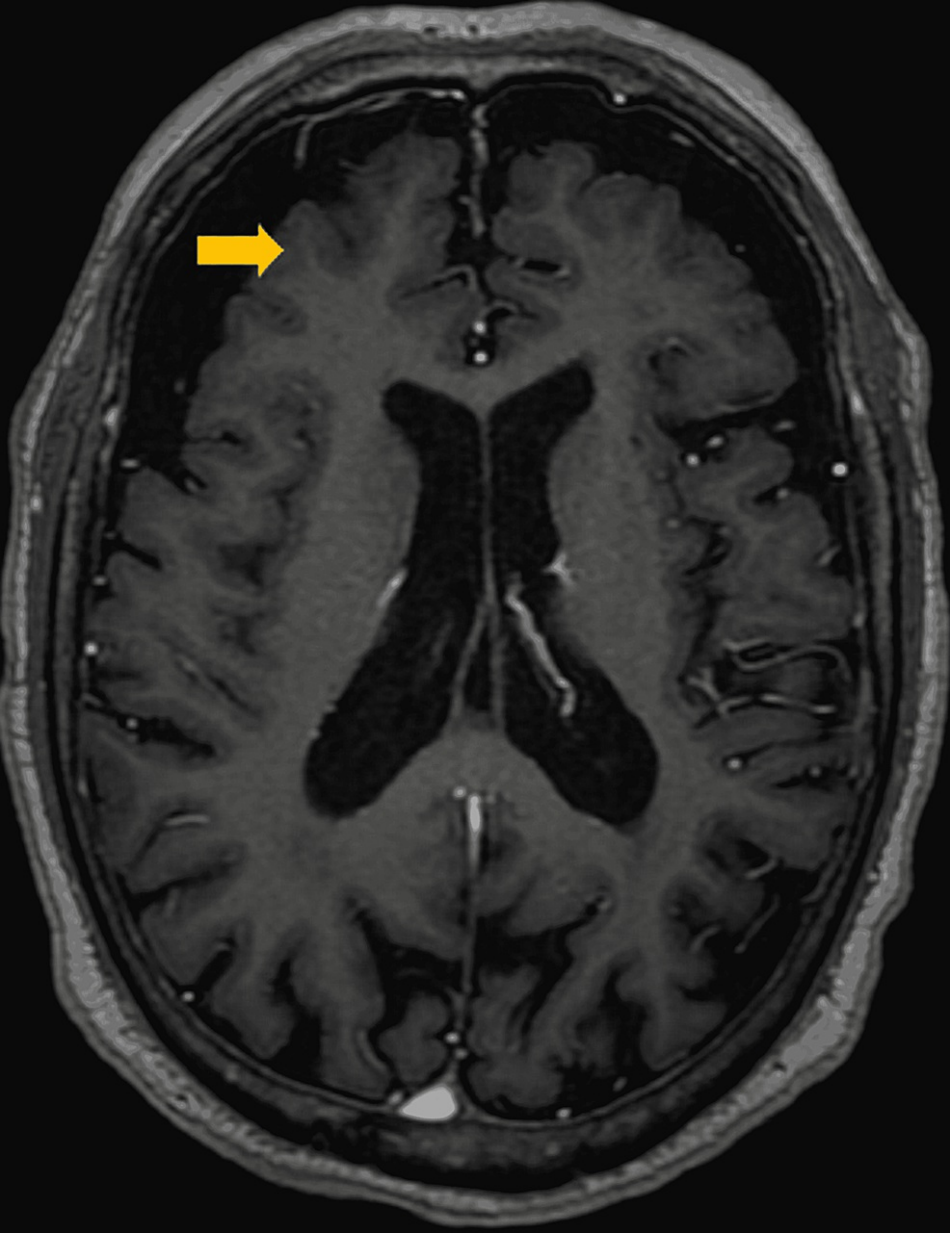
Magnetic resonance imaging of the brain illustrates cortical and subcortical atrophy with mild compensatory ventricular ectasia

**Figure 2:**
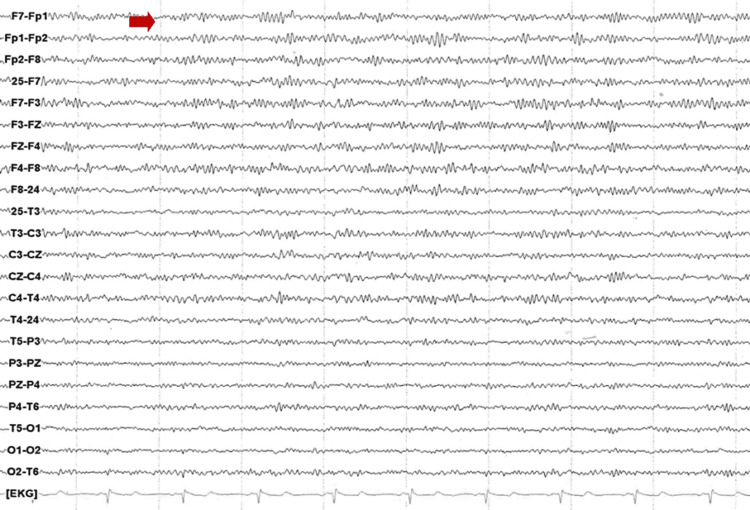
Electroencephalogram of the patient shows low unstable alpha activity as well as excess fast beta activity

The patient was administered 1mg orally of Lorazepam upon his arrival at the psychiatric emergency department, since at that time he was presenting an episode with the aforementioned characteristics. His stay in said service was approximately an hour and a half, and during this period he did not present other paroxysms. Initially, a primary psychiatric disorder was not diagnosed, and it was decided to continue the same pharmacological treatment to allow an adequate response margin since the patient had been receiving treatment for approximately one month. The patient was referred to the clinical services department for further outpatient symptom management and care due to suspicion that the patient's symptoms appeared to be neuropsychiatric or frankly neurological in nature. A few days after said care, a non-contrasted magnetic resonance imaging of the brain was performed, which reported cortical and subcortical atrophy with mild compensatory ventricular ectasia as well as deviation of the nasal septum to the right with a contacting bone spur (Figure 1). Likewise, a conventional electroencephalogram was performed, which was reported as moderately abnormal due to low unstable alpha activity, excess fast beta activity, as well as non-specific findings suggestive of diffuse cortical and subcortical alterations (Figure 2). The rest of his laboratory tests were unaltered, and an epileptiform diagnosis rather than a primary psychiatric one was suspected due to these findings. His pharmacological treatment was changed to Sertraline 50mg/day and Pregabalin 150mg/night, with which the patient reported slight improvement in the frequency and duration of the paroxysms.

The patient continued his neuropsychiatric care at the institution, regularly attending subsequent consultations, but to date, he reported that he persists with the symptoms already described, without presenting total remission of these, and that he continues to experience dysfunction in his daily activities due to them. During this period, the patient attended the psychiatric emergency service a further five times, reporting in each of these that the paroxysms did not subside despite treatment and stating a desire to continuously adjust his antiepileptic medication hoping to alleviate the daily disfunction derived from the DS. Psychiatric symptoms were ruled out on all occasions in these assessments, no other tonic, clonic, myoclonic or absence seizures were found, and the patient resumed his conventional follow-up consultations. Additionally, the patient reaffirmed maintaining adherence to his pharmacological treatment and consultations despite what has already been mentioned. Interestingly, he denied having experienced any adverse effects associated with taking the medication. Finally, the patient gave his written informed consent after being informed of the interest in preparing this case report due to the uniqueness of his clinical picture.

## Discussion

This case report illustrates a middle-aged individual with no previous neuropsychiatric history who abruptly began to present stereotyped and paroxysmal crying. These episodes seemed to be accompanied by an aura due to referred abdominal discomfort, occurring intermittently but often enough during the day to cause general dysfunction. For this reason, the patient received psychiatric care due to the suspicion of a mental health problem derived from the uncontrollable crying that he presented. However, the absence of psychiatric symptoms before, during, and after the onset of his condition is striking, except for anxious subjective discomfort derived from the consequences of these paroxysms. Additionally, although the patient did not receive a formal psychiatric diagnosis, he did receive conventional pharmacological treatment which did not result in the lasting improvement of his symptoms. For this reason, he went to a psychiatric emergency service, possibly desperate due to the non-remission of his illness, where it was considered to postpone a primary psychiatric diagnosis due to the suspicion of a neurological one after a more concise assessment regarding his history, clinical picture, and physical condition at the time of the assessment. During follow-up, abnormal findings were found on his cerebral imaging and electroencephalogram, suggestive of a nonspecific epileptiform syndrome, which is why his pharmacological treatment was changed to one with a more favorable profile to address these findings. The patient did identify slight improvement with this medication adjustment, but to date, he persists with the paroxysms outlined above.

The findings described in this report are relevant because, although the results of neurophysiological tests and brain imaging were not highly specific, they are abnormal enough to suspect a diagnosis of DS based on the patient's symptoms. Furthermore, these results are consistent with what has already been reported in the literature [2]. If anything, the only element that could be studied further is whether the patient really does not have a hypothalamic hamartoma, a common finding in previous reports, although the magnetic resonance that was performed apparently rules it out [8]. The refractoriness of symptoms to antiepileptic treatment is also in line with what has been previously reported in the literature [3]. For instance, it is thought that surgical treatment might be an effective therapy for DS, assuming that they occur in cases where a hypothalamic hamartoma is identified. Though, in cases where such a lesion is not detected, it has been suspected that calcium channel antagonism might be helpful [3]. Perhaps this would explain why the patient partially responded to the use of a gabapentinoid such as Pregabalin, which finally acts through the modulation of alpha-2-delta channels, which are calcium-dependent [14]. However, to date, it is unknown how much the patient can improve with an optimized treatment since there is no consensus on the best therapy for DS.

The strength of this report is due to the fact that it adds value to the medical literature since it reinforces the findings that have been previously described, not only in terms of symptoms, their evolution, management, and partial response to treatment but also alerts the psychiatric union that this type of ailment could inadvertently fall into their care. This would force psychiatrists or personnel who treat mental health to be attentive when evaluating unusual emotional symptoms. The limitations of this work lie in the weaknesses of case reports. These are unable to illustrate direct causality between the phenomena being described, and of course, lack in-depth statistical analysis or scrutiny of what is found. It would be interesting to see the results of a cohort with an acceptable number of participants, or studies comparing different antiepileptic treatments to start a debate about a consensus on the treatment of these individuals. For example, a prospective study could make it possible to follow this population and assess more precisely if there is any change in its prognosis. On the other hand, an analytical study could pave the way to measure the differences in a more specific treatment, such as the use of gabapentinoids, or any other antiepileptic drug. More importantly for this case, observational studies of any nature focused on the psychiatric aspects of these patients could provide tools to timely identify these symptoms in case they reach the hands of mental health personnel. Nevertheless, the merit of this manuscript lies in the academic dissemination of DS, motivating anyone interested in neuroscience to continue their study.

## Conclusions

DS are unusual epileptiform phenomena due to their rarity and the characteristics of their presentation. Paroxysms distinguished by uncontrollable crying could easily be mistaken for psychiatric symptoms, when in fact they could be demonstrating an alteration in cerebral electrical rhythmicity. In the case of this patient, the DS were detected in a timely manner, despite the fact that the individual went to a psychiatric emergency service. During his evolution and follow-up, pharmacological adjustments were made that did not completely alleviate the patient's symptoms. This is in line with what is reported in the literature, which describes that these phenomena are often refractory to treatment. However, the present work has the merit of corroborating some aspects already reported in the neuropsychiatric literature on this kind of seizure, and the neuroscientific guild is encouraged to continue their study.

## References

[1] Luciano D, Devinsky O, Perrine K (1993). Crying seizures. Neurology.

[2] Hogan RE, Rao VK (2006). Hemifacial motor and crying seizures of temporal lobe onset: case report and review of electro-clinical localisation. J Neurol Neurosurg Psychiatry.

[3] Helen Cross J, Spoudeas H (2017). Medical management and antiepileptic drugs in hypothalamic hamartoma. Epilepsia.

[4] Wortzel HS, Oster TJ, Anderson CA, Arciniegas DB (2008). Pathological laughing and crying: epidemiology, pathophysiology and treatment. CNS Drugs.

[5] Kanner AM (2013). Dacrystic seizures: Do they have any localizing value?. Epilepsy Behav.

[6] Oehl B, Brandt A, Fauser S, Bast T, Trippel M, Schulze-Bonhage A (2010). Semiologic aspects of epileptic seizures in 31 patients with hypothalamic hamartoma. Epilepsia.

[7] Tatum WO, Loddenkemper T (2010). Crying with left temporal lobe seizures and Wada testing. Epilepsy Behav.

[8] De La Mota CC, Del Valle FM, Villena AP, Gero MC, Del Pozo RL, Rojas MR (2012). Hypothalamic hamartoma in paediatric patients: clinical characteristics, outcomes and review of the literature. Neurologia.

[9] Blumberg J, Fernández IS, Vendrame M (2012). Dacrystic seizures: demographic, semiologic, and etiologic insights from a multicenter study in long-term video-EEG monitoring units. Epilepsia.

[10] Gadoth A, Singh J, Britton JW, Flanagan EP, Pittock SJ (2017). Dacrystic seizures-a cry for help. Neurol Neuroimmunol Neuroinflamm.

[11] Jin B, Wu H, Xu J (2014). Analyzing reliability of seizure diagnosis based on semiology. Epilepsy Behav.

[12] Teche SP, Laporte P, Medeiros F, Braggati JA, Gomes F (2015). Dacrystic seizures and psychogenic non-epileptic seizures: a case report. J Neurol Disord.

[13] Riley DS, Barber MS, Kienle GS (2017). CARE guidelines for case reports: explanation and elaboration document. J Clin Epidemiol.

[14] Uchitel OD, Di Guilmi MN, Urbano FJ, Gonzalez-Inchauspe C (2010). Acute modulation of calcium currents and synaptic transmission by gabapentinoids. Channels (Austin).

